# May anti‐seizure medications alter brain structure in temporal lobe epilepsy? A prospective study

**DOI:** 10.1002/epi4.12912

**Published:** 2024-03-12

**Authors:** Ilaria Sammarra, Maria Eugenia Caligiuri, Maria Celeste Bonacci, Gianfranco Di Gennaro, Francesco Fortunato, Iolanda Martino, Alessia Giugno, Angelo Labate, Antonio Gambardella

**Affiliations:** ^1^ Department of Medical and Surgical Sciences, Institute of Neurology Magna Græcia University of Catanzaro Catanzaro Italy; ^2^ Neuroscience Research Center, Department of Medical and Surgical Sciences Magna Græcia University of Catanzaro Catanzaro Italy; ^3^ Department of Medical and Surgical Sciences Magna Græcia University of Catanzaro Catanzaro Italy; ^4^ Department of Health Sciences Magna Græcia University of Catanzaro Catanzaro Italy; ^5^ Neurophysiopatology and Movement Disorders Clinic University of Messina Messina Italy

**Keywords:** anti‐seizures medications (ASMs), cerebral gray matter, mesial temporal lobe epilepsy (MTLE), neuroimaging

## Abstract

Mild mesial temporal lobe epilepsy (MTLE) patients may remain untreated for a considerable time after disease onset or achieve seizure control with a single anti‐seizures medication (ASM). Thus, they represent an optimal population to investigate whether ASMs might have influence on brain structure. We consecutively enrolled 56 mild MTLE patients (22/56 untreated, 34/56 on‐monotherapy) and 58 healthy controls, matched for age and gender. All subjects underwent 3T‐brain MRI, using FreeSurfer for automated morphometry. Differences in gray matter were assessed using one‐way Analysis of Covariance (ANCOVA), adjusting for age, disease duration and intracranial volume. No significant change was observed between treated and untreated patients. We observed a significant reduction in cortical thickness of left inferior parietal, inferior temporal, middle temporal gyri, and right inferior parietal gyrus, temporal pole in monotherapy patients compared to healthy controls, as well as an increase in left isthmus of cingulate gyrus in untreated MTLE subjects compared to controls. Surface and subcortical volumes analysis revealed no differences among groups. Our study demonstrated no substantial morphological abnormalities between untreated mild MTLE patients and those undergoing monotherapy. Although exploratory, these results may reassure about safety of commonly used drugs and their marginal role in influencing neuroimaging results.

**Plain Language Summary:**

This study investigated the following question: can medications against epileptic seizures have an effect on brain structure in mild mesial temporal lobe? Preliminary results from our analyses suggest not, as we did not find any difference in brain gray matter between untreated patients and those treated with a single anti‐seizures medication. On the other hand, epilepsy patients presented cortical thinning compared to healthy controls in several regions of the temporal and parietal lobes, in line with previous studies investigating the disease.

**Key points**

- Regardless of ASMs’ assumption, mild MTLE patients presented cortical thinning in temporal and parietal lobes compared with healthy subjects.
- No significant structural brain changes were observed between untreated and on‐monotherapy mild MTLE subjects.
- Lack of significant grey matter changes associated with ASMs suggest commonly used therapies may have a marginal influence on neuroimaging findings.

## 1 INTRODUCTION

In existing literature on epileptic syndromes, the administration of one or more anti‐seizure medications (ASMs) has been considered a potential confounding factor for neuroimaging findings regarding brain structure and function.^1^ However, to what extent ASMs may influence brain morphology remains unknown, preventing the possibility to univocally associate the epileptic syndrome itself with any change detected using magnetic resonance imaging (MRI) techniques. In particular, cerebellar degeneration has been correlated to chronic phenytoin exposure,^2^ whereas valproate has been associated with reduced parietal thickness, total gray, and white matter volumes.^3^ Moreover, transient brain volume changes were demonstrated in animal studies after injection of valproate or levetiracetam.^4^ On the other hand, levetiracetam revealed a normalizing effect in terms of increased cortical thickness in the Rolandic regions of children with Childhood Epilepsy with Centrotemporal Spikes (CECTS)^5^ as well as a mild significant improvement in cognitive abilities in the same group of patients.^6^

Studying the impact of medications on brain structures requires the comparison between groups of patients affected by the same epileptic syndrome, with and without antiseizure therapy.^5^ However, these cohorts cannot be easily enrolled, considering how difficult it is to identify treated and untreated patients with comparable clinical features, such as disease duration.

In the light of these considerations, patients with mild mesial temporal lobe epilepsy (MTLE) represent the optimal population to enroll to overcome the abovementioned problem. Indeed, mild MTLE patients tend to remain untreated for a considerable time, thanks to their relatively benign symptomatology (e.g., viscerosensory auras), and even when treated, they easily reach seizure freedom with a single ASM.^7^, ^8^, ^9^ This study had the exploratory and hypothesis‐generating aim to analyze brain morphometry changes in healthy controls and mild MTLE patients grouped according to their history of medication: patients with up to 10 years of monotherapy versus untreated patients with comparable disease duration. Furthermore, we investigated the possible presence of association between gray matter changes and demographic and clinical variables.

## 2 MATERIALS AND METHODS

### 2.1 Subjects

Demographic and clinical data are summarized in Table 1. From March 2018 to April 2021, we consecutively enrolled 56 patients affected by mild MTLE (34 females; 37.9 ± 13 years) undergoing routine follow‐up at our Outpatients Epilepsy Clinics. The diagnosis of mild MTLE was made if patients were either completely seizure‐free or had nondisabling auras for at least 24 months, with or without appropriate ASM.^7^ Thirty‐four of 56 were taking one ASM (19 females; 36.6 ± 12.3 years), while the remaining 22 patients (15 females; 39.8 ± 14.2 years) had not started any treatment because of the relatively benign nature of the symptoms, causing a subsequent diagnostic delay of various years.^8^, ^9^, ^10^

**TABLE 1:** Demographic and clinical characteristics of epileptic subjects and healthy controls.

	Untreated mild MTLE	Monotherapy mild MTLE	Healthy controls	*p*‐value
	*n* = 22	*n* = 34	*n* = 58	
Age, years	39.8 ± 14.2	36.6 ± 12.3	37.2 ± 10.6	0.587^a^
Gender, *n*° (% female)	15/22 (68.2%)	19/34 (55.8%)	28/58 (48.3%)	0.274^b^
Epilepsy duration, years	5.8 ± 8.1	9.9 ± 9	‐	0.085^c^
Family history of FS/epilepsy, *n*° (%)	4/22 (18.2%)	9/34 (26.5%)	‐	0.535^d^
Personal history of FS, *n* (%)	2/22 (9%)	6/34 (17.6%)	‐	0.47^d^
Interictal EEG, *n*° (%)				
Unilateral left	10/22 (45.5%)	18/34 (52.9%)	‐	
Unilateral right	4/22 (18.2%)	5/34 (14.7%)		
Bilateral	3/22 (13.6%)	10/34 (29.4%)		
Normal	5/22 (22.7%)	1/34 (2.9%)		
HS side, *n*° (%)				
Left	1/22 (4.5%)	3/34 (8.8%)	‐	
Right	1/22 (4.5%)	2/34 (5.9%)		
Bilateral	1/22 (4.5%)	1/34 (2.9%)		
None	19/22 (86.4%)	28/34 (82.4%)		
AMSs duration intake, years	‐	6.1 ± 5	‐	

Abbreviations: AMS, anti‐seizure medications; EEG, electroencephalography; FS, febrile seizures; HS, hippocampal sclerosis; MTLE, mesial temporal lobe epilepsy.

^a^ Kruskal‐Wallis test.

^b^ Fisher's exact test 3 × 2 contingency table.

^c^ Unpaired *t*‐test.

^d^ Fisher's exact test.

Additional clinical features included onset in adulthood or late adolescence, normal neurological and cognitive examination, unremarkable past medical history.^8^, ^9^, ^10^ Exclusion criteria were as follows: (1) polytherapy and history of other previous anti‐seizure treatments, to limit potentially confounding effects of multiple ASMs; (2) presence of epileptogenic lesions on MRI different from hippocampal sclerosis (HS) (i.e., tumors, cerebrovascular disease, malformations of cortical development, post‐traumatic scars). Evidence of HS, defined by the characteristic MRI pattern of atrophy on T1‐weighted and hyperintensity on T2‐weighted or FLAIR MRI,^10^ was not considered an exclusion factor, as it has been observed in up to one third of mild MTLE patients.^7^

Fifty‐eight healthy controls (28 females; 37.2 ± 10.6 years) matched for age and sex were also included. Inclusion criteria were (1) no history of neurological disease, (2) unremarkable neurological examination. Local research ethics committee approval was obtained, and all participants gave written informed consent to the study.

### 2.2 MRI acquisition and analysis

All subjects underwent 3T‐brain MRI on a Discovery MR‐750 GE scanner with an eight‐channel head coil. The Harmonized Neuroimaging of Epilepsy Structural Sequences (HARNESS‐MRI) protocol^11^ was acquired, including a 3D T1‐weighted spoiled gradient echo sequence (sagittal acquisition, TE/TR = 3.7/9.2 ms, matrix size 256 × 256, flip angle = 12°, isotropic voxel = 1 × 1 × 1 mm^3^). *FreeSurfer* (v 7.2) (http://surfer.nmr.mgh.harvard.edu) was used to perform automated brain morphometric analysis and obtain, using the standard pipeline^12^, ^13^ the following measures: thickness from 34 gray matter cortical regions for each hemisphere, as well as 14 subcortical volumes: left and right hippocampus, amygdala, caudate, nucleus accumbens, pallidum, putamen, and thalamus.^12^

### 2.3 Statistical analysis

The normal distribution of each variable was assessed through Shapiro–Wilk test and visual inspection of histograms, Q‐Q plots, and box plots. Data were considered normally distributed for *p*‐value above 0.05. For continuous variables, mean and standard deviation or median and interquartile range were reported for normally and not normally distributed values, respectively, as well as unpaired *t*‐test and Kruskal–Wallis test were used to assess differences between groups. Categorical variables, expressed as frequencies and percentages, were compared through Fisher's exact test 3 × 2 contingency table. Differences in cortical and subcortical gray matter between naïve and on‐treatment mild MTLE patients compared to healthy controls were assessed using one‐way Analysis of Covariance (ANCOVA), adjusting for possible clinical confounders as age, disease duration and intracranial volume. The Bonferroni post‐hoc test was used in order to examine pairwise differences. Levene's test and normality checks were carried out and the assumptions met. Statistical significance was set at 5%. Statistical analysis was performed using IMB Statistical Package for Social Science software (SPSS, version 26.0, Chicago, IL, USA) for Windows.

## 3 RESULTS

### 3.1 Patients' clinical features

Clinical data are reported in table section (Table 1). There was no significant difference in mean age at MRI scan (*F*[2.111] = 0.535; *p* = 0.587) and in sex (*χ* ^2^ = 2.59, *p* = 0.274) between untreated and on‐treatment mild MTLE patients compared to healthy controls. The untreated mild MTLE group had low seizure frequency, and the on‐treatment patients were seizure free taking a single medication. Specifically, in the on‐treatment mild MTLE group, 12 out of 34 patients were taking carbamazepine, 7/34 lamotrigine, 1/34 oxcarbazepine, 7/34 levetiracetam, 5/34 topiramate and, 2/34 valproate. Although the disease duration was longer in treated patients (9.9 ± 9 years) compared to untreated mild MTLE subjects (5.8 ± 8.1 years), this difference was not statistically significant (*t*[48.3] = −1.76; *p* = 0.085). Moreover, frequency of family history of epilepsy and febrile convulsions (FCs), as well as personal history of FCs, were similar in the two mild MTLE groups.

### 3.2 Cortical and subcortical gray matter analysis results

In all mild MTLE patients compared to controls, ANCOVA revealed significant thinning of left inferior parietal [*F*(1,109) = 6.84; *p* = 0.010; Cohen's *f*: 0.80], inferior temporal [*F*(1,109) = 5.79; *p* = 0.018; Cohen's *f*: 0.30] and middle temporal gyri [*F*(1,109) = 5.77; *p* = 0.018; Cohen's *f*: 0.27] paralleled by increased thickness in isthmus of left cingulate gyrus [*F*(1,109) = 6.56; *p* = 0.012; Cohen's *f*: 0.95]. No significant changes were demonstrated in surface and subcortical volumes analysis.

**FIGURE 1:**
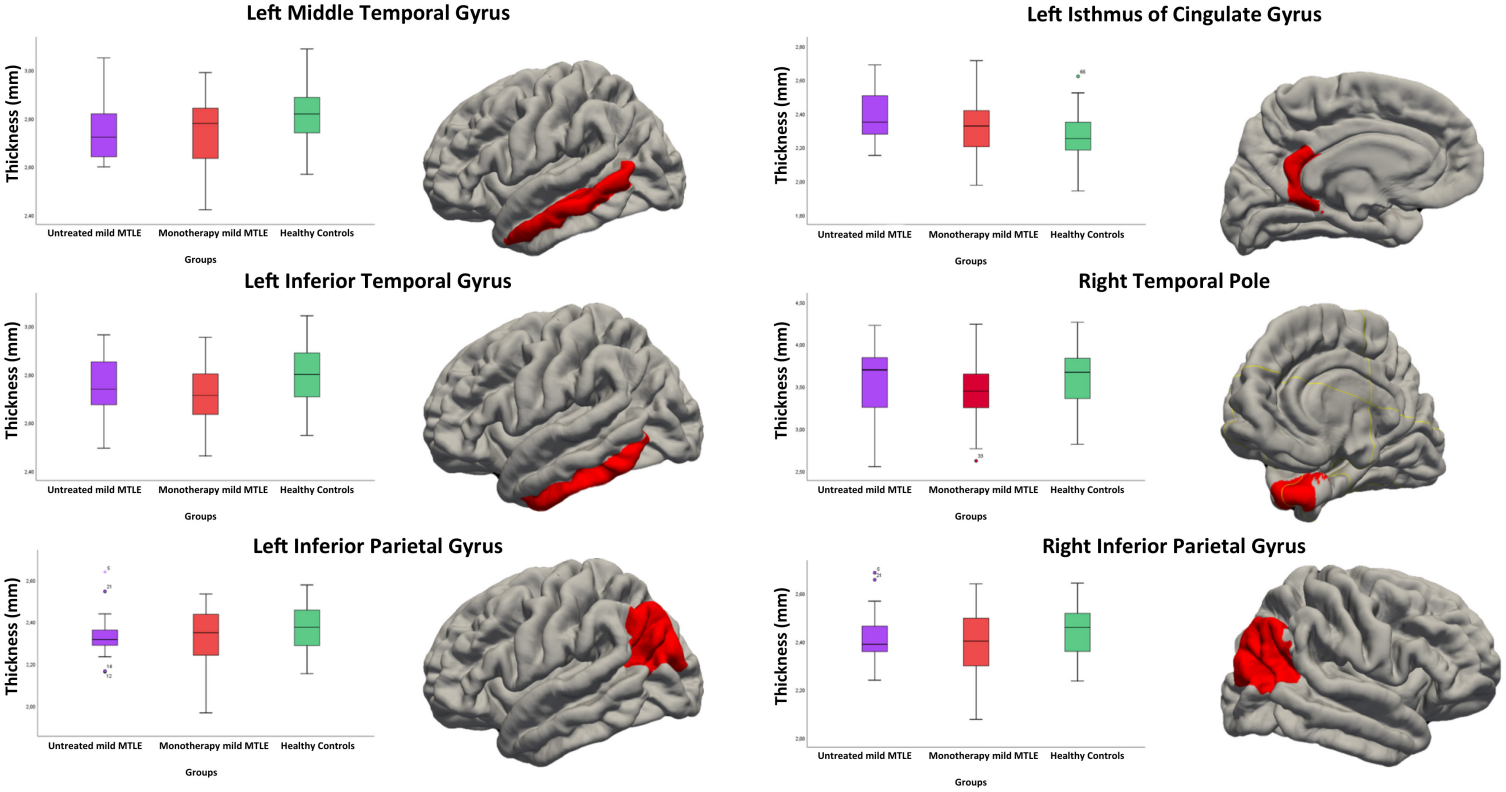
Box plots showing distribution of variables demonstrating significant differences in cortical thickness between subgroups of mild MTLE and healthy controls (significant regions are depicted in red on the cortical surface).

As shown in Figure 1, when performing ANCOVA among three groups (controls, treated and untreated mild MTLE), we observed significant differences in cortical thickness of left inferior parietal [*F*(2,108) = 3.84; *p* = 0.025; Cohen's *f*: 0.20], left inferior temporal [*F*(2,108) = 4.09; *p* = 0.019; Cohen's *f*: 0.32], middle temporal gyri [*F*(2,108) = 3.59; *p* = 0.031; Cohen's *f*: 0.27], right inferior parietal gyrus [*F*(2,108) = 3.29; *p* = 0.041; Cohen's *f*: 0.20], temporal pole [*F*(2,108) = 3.39; *p* = 0.037; Cohen's *f*: 0.20]as well as in left isthmus of cingulate gyrus [*F*(2,108) = 6.17; *p* = 0.019; Cohen's *f*: 0.33]. As reported in table section (Table 2), post‐hoc tests indicated cortical thinning in monotherapy patients compared to healthy controls concerning left inferior parietal (*p* = 0.023), left inferior temporal (*p* = 0.015), middle temporal gyri (*p* = 0.026), in left pars orbitalis (*p* = 0.033), in right inferior parietal gyrus (*p* = 0.037) and temporal pole (*p* = 0.034). Untreated subjects showed no change in the abovementioned structures comparing to monotherapy patients as well as to healthy controls. Moreover, we found a significant cortical increase of left cingulate isthmus in untreated mild MTLE patients compared to healthy controls (*p* = 0.001), without no statistical results in comparing monotherapy patients to healthy controls and on‐treatment patients to untreated ones. Thickness changes in these abovementioned regions did not correlate with disease duration. Among these three groups, no significant difference was demonstrated in surface and subcortical volumes analysis.

**TABLE 2:** Cortical thickness comparisons among groups using ANCOVA.

Cortical thickness					
Brain region	*p*‐value	Bonferroni adjusted pairwise comparisons			
		Groups	Mean ± SD	Comparison	*p*‐value
Left isthmus cingulate gyrus	0.002***	Monotherapy MTLE	2.327 ± 0.17	Healthy controls	0.932
		Untreated MTLE	2.387 ± 0.16	Monotherapy MTLE	0.062
		Healthy controls	2.258 ± 0.14	Untreated MTLE	0.002***
Left inferior parietal gyrus	0.025**	Monotherapy MTLE	2.329 ± 0.14	Healthy controls	0.023**
		Untreated MTLE	2.332 ± 0.11	Monotherapy MTLE	1.000
		Healthy controls	2.377 ± 0.11	Untreated MTLE	0.266
Left inferior temporal gyrus	0.019**	Monotherapy MTLE	2.713 ± 0.13	Healthy controls	0.015**
		Untreated MTLE	2.748 ± 0.12	Monotherapy MTLE	0.391
		Healthy controls	2.8 ± 0.13	Untreated MTLE	0.661
Left middle temporal gyrus	0.031*	Monotherapy MTLE	2.751 ± 0.14	Healthy controls	0.026*
		Untreated MTLE	2.755 ± 0.13	Monotherapy MTLE	0.722
		Healthy controls	2.819 ± 0.12	Untreated MTLE	0.489
Right inferior parietal gyrus	0.041*	Monotherapy MTLE	2.397 ± 0.12	Healthy controls	0.037*
		Untreated MTLE	2.418 ± 0.11	Monotherapy MTLE	0.326
		Healthy controls	2.447 ± 0.1	Untreated MTLE	1.000
Right temporal pole	0.037*	Monotherapy MTLE	3.418 ± 0.38	Healthy controls	0.034*
		Untreated MTLE	3.569 ± 0.44	Monotherapy MTLE	0.293
		Healthy controls	3.637 ± 0.33	Untreated MTLE	1.000

*Note*: The value strength of significant post‐hoc *p*‐values are classified as following: *0.025 ≤ *p* > 0.05; **0.01 ≤ *p* < 0.025; ***p < 0.01.

Abbreviation: MTLE, mesial temporal lobe epilepsy.

Group‐wise analyses were repeated for the ipsilateral/contralateral side in those patients having a clear lateralization of epileptiform discharges on EEG recordings (14/22 untreated mild MTLE patients and 22/34 on‐monotherapy subjects, as shown in Table 1). Results were essentially unchanged compared to those obtained with left and right sides and are reported in Table S1.

## 4 DISCUSSION

In this exploratory work, we found no morphological differences between untreated patients with mild MTLE and those undergoing monotherapy without considering ASMs' mechanism of action, through a comprehensive evaluation of structural MRI. Our results support the hypothesis that ASMs may not cause any significant change in brain structure of these patients, characterized by long‐term seizures freedom and similar disease duration. We also found cortical thinning of left temporal and parietal areas in all mild MTLE patients and in monotherapy mild MTLE compared to controls, observing medium to high effect sizes, as measured by Cohen's *d*, in agreement with our previous studies investigating mild phenotype.^10^

In detail, mild MTLE patients with HS compared to healthy controls demonstrated a significant thinning of left and right precentral and postcentral gyri, superior, middle, and inferior prefrontal cortex, supramarginal gyrus, and occipital lobe. The MRI‐negative mild MTLE group showed an overlapping but less prominent thinning pattern.^10^ These results are also in line with the larger multicentre ENIGMA‐Epilepsy study, where a small‐to‐moderate effect size was reported for gray matter abnormalities in the general MTLE population. In particular, the HS‐positive population showed reduced thickness involving motor regions (i.e., precentral and postcentral gyri), frontal cortex (i.e., superior and middle frontal gyri), and mesiotemporal regions (i.e., temporopolar cortex and parahippocampal gyrus). Concomitantly, the MRI‐negative MTLE group demonstrated a similar pattern of structural abnormalities with a less prominent involvement of temporal regions.^1^

Understanding the influence of ASMs on the brain of epilepsy patients still represents an unsolved research question. Indeed, despite several studies reported transient atrophy of parietal areas, total gray, and white matter volumes^3^ as well as occipital cortices^14^ in subjects exposed to valproate, in vitro studies demonstrated a neuroprotective role of this ASM.^15^ Uncertainty regarding ASMs role on reported literature findings is likely due to difficulties in compensating for the effects of drug‐resistance in clinical cohorts and enrolling simultaneously on‐treatment and untreated epileptic patients with a sufficiently long disease duration. For this reason, in this study we enrolled a population of mild MTLE, which allowed to limit the influence of seizure recurrence and number of ASMs on detected changes, thanks to its unique clinical characteristics, i.e., subtle symptoms that can be frequently misdiagnosed, and consequently patients can remain untreated for a considerable time. Indeed, we were able to enroll untreated patients with a comparable disease history to those on‐treatment.

Interestingly, we found increased thickness of left cingulate gyrus's isthmus in untreated patients compared to healthy controls, but not in those undergoing monotherapy. The posterior cingulate cortex, a crucial node of the default mode network, is connected to the parahippocampal region through the isthmus. In TLE, it has been associated with memory impairment by using functional MRI.^16^ A possible explanation for this increase, which may seem counterintuitive, can be found in a possible “normalizing” effect of antiepileptic therapy on cingulate cortex. Indeed, previous work suggested a remodeling role of ASMs on brain structure. For instance, levetiracetam contributed to stabilization of abnormal cortical thickness and cognitive performance improvement in children with CECTS.^5^, ^6^ Beneficial effects of levetiracetam were also associated with progressive improvement of visuo‐spatial abilities in TLE patients.^17^

There are of course limitations to our study. First, our work was purely exploratory with a relatively small number of enrolled subjects, due to our very restrictive inclusion criteria and need of minimizing difference in disease duration between subgroups. Partially related to the previous point, the limited number of subjects prevented stratification of patients according to action mechanisms of different ASMs. Another limitation could be considered the absence or low number of ASMs taken by the patients previously demonstrated as influencing brain morphology, such as phenytoin and valproate.^2^, ^3^ Further analyses conducted on a wider sample and subsequent categorization for ASMs' type will contribute to better clarify our current results and characterize the impact of these drugs on gray and eventually white matter modifications.

Overall, our study contributes to delineating the role of ASMs on brain structure, in a study cohort including patients with up to 10 years of medication history. The substantial lack of significant ASMs effect on cerebral gray matter reassures about the safety of most used drugs in focal epilepsy and on their potentially marginal role in influencing neuroimaging results.

## CONFLICT OF INTEREST STATEMENT

None of the authors has any conflict of interest to disclose. We confirm that we have read the Journal's position on issues involved in ethical publication and affirm that this report is consistent with those guidelines.

## ETHICS STATEMENT

This study has been approved by our Institution's Ethics Committee and has been performed in accordance with the ethical standards laid down in the 1964 Declaration of Helsinki and its later amendments.

## PATIENT CONSENT STATEMENT

A written informed consent was obtained from all patients enrolled.

## Supporting information


Table S1

## Data Availability

The authors are available to share clinical and experimental data used in this study with other interested investigators for addressing specific hypotheses for future studies.
