# Supplementary material for: May anti‐seizure medications alter brain structure in temporal lobe epilepsy? A prospective study
Source: Epilepsia Open. 2024 Mar 12;9(3):1076–82. doi: 10.1002/epi4.12912 (PMC11145604; doi:10.1002/epi4.12912)
Supplement: Supplementary file 1 — Table S1 [file EPI4-9-1076-s001.docx]

| Cortical thickness | | | | | |
| --- | --- | --- | --- | --- | --- |
| Brain region | p-value | Bonferroni adjusted pairwise comparisons | | | |
|  |  | Groups | Mean ± SD | Comparison | p-value |
| Ipsilateral isthmus cingulate gyrus | 0.014****** | Monotherapy MTLE | 2.33±0.2 | Healthy Controls | 0.468 |
|  |  | Untreated MTLE | 2.37±0.2 | Monotherapy MTLE | 0.413 |
|  |  | Healthy Controls | 2.26±0.1 | Untreated MTLE | 0.011****** |
| Ipsilateral middle temporal | 0.015****** | Monotherapy MTLE | 2.75 ± 0.1 | Healthy Controls | 0.013****** |
|  |  | Untreated MTLE | 2.75 ± 0.1 | Monotherapy MTLE | 0.762 |
|  |  | Healthy Controls | 2.82 ± 0.1 | Untreated MTLE | 0.284 |
| Ipsilateral rostral middle frontal | 0.019****** | Monotherapy MTLE | 2.39 ± 0.1 | Healthy Controls | 0.018****** |
|  |  | Untreated MTLE | 2.38 ± 0.1 | Monotherapy MTLE | 0.995 |
|  |  | Healthy Controls | 2.43 ± 0.1 | Untreated MTLE | 0.247 |
| Contralateral inferior parietal | 0.006******* | Monotherapy MTLE | 2.39 ± 0.1 | Healthy Controls | 0.005******* |
|  |  | Untreated MTLE | 2.40 ± 0.1 | Monotherapy MTLE | 0.591 |
|  |  | Healthy Controls | 2.45 ± 0.1 | Untreated MTLE | 0.207 |
| Contralateral inferior temporal | 0.010****** | Monotherapy MTLE | 2.71 ± 0.2 | Healthy Controls | 0.007****** |
|  |  | Untreated MTLE | 2.75 ± 0.2 | Monotherapy MTLE | 0.195 |
|  |  | Healthy Controls | 2.81 ± 0.1 | Untreated MTLE | 0.803 |

MTLE, mesial temporal lobe epilepsy.

The value strength of significant post-hoc p-values are classified as following:

*0.025≤p<0.05

**0.01≤p<0.025

***p<0.01
